# Supplementary material for: Delta-8 Tetrahydrocannabinol Exposures Reported to US Poison Centers: Variations Among US States and Regions and Associations with Public Policy
Source: J Med Toxicol. 2024 Aug 21;20(4):389–400. doi: 10.1007/s13181-024-01030-z (PMC11436591; doi:10.1007/s13181-024-01030-z)
Supplement: Supplementary file 1 — Supplementary Material 1: Appendix 1. Categorization of States by United States Region. Appendix 2. Categorization of States by Status of State Regulation of Delta-8 THC. Appendix 3. Categorization of States by Status of State Legislation on Cannabis (Delta-9 THC). Appendix 4. Rate of Exposures Involving Delta-8 THC Reported to the National Poison Data System by United States Region and by Year, 2021–2022. Appendix 5. Rate of Exposures Involving Delta-8 THC Reported to the National Poison Data System by Year and Status of State Regulation of Delta-8 THC, 2021–2022. Appendix 6. Rate of Exposures Involving Delta-8 THC Reported to the National Poison Data System by Year and Status of State Cannabis (Delta-9 THC) Legalization, 2021–2022. [file 13181_2024_1030_MOESM1_ESM.docx]

| **Appendix 1. Categorization of States by United States Region** | | | |
| --- | --- | --- | --- |
| **United States Region** | | | |
| **Northeast** | **Midwest** | **South** | **West** |
| Connecticut | Illinois | Alabama | Alaska |
| Maine | Indiana | Arkansas | Arizona |
| Massachusetts | Iowa | Delaware | California |
| New Hampshire | Kansas | District of Columbia | Colorado |
| New Jersey | Michigan | Florida | Hawaii |
| New York | Minnesota | Georgia | Idaho |
| Pennsylvania | Missouri | Kentucky | Montana |
| Rhode Island | Nebraska | Louisiana | Nevada |
| Vermont | North Dakota | Maryland | New Mexico |
|  | Ohio | Mississippi | Oregon |
|  | South Dakota | North Carolina | Utah |
|  | Wisconsin | Oklahoma | Washington |
|  |  | South Carolina | Wyoming |
|  |  | Tennessee |  |
|  |  | Texas |  |
|  |  | Virginia |  |
|  |  | West Virginia |  |

Source: US Census Bureau <https://www2.census.gov/geo/pdfs/maps-data/maps/reference/us_regdiv.pdf> [15].

| **Appendix 2. Categorization of States by Status of State Regulation of Delta-8 THC** | | |
| --- | --- | --- |
| **Delta-8 THC Regulation Status** | | |
| **Delta-8 THC Unregulated** | **Delta-8 THC Banned** | **Delta-8 THC Restricted** |
| Alabama | Alaska | California |
| Arizona | Colorado | Connecticut |
| Arkansas | Delaware | Iowa |
| District of Columbia | Hawaii | Kansas |
| Florida | Idaho | Louisiana |
| Georgia | Massachusetts | Maryland |
| Illinois | Montana | Michigan |
| Indiana | Nevada | South Dakota |
| Kentucky | New York | Virginia |
| Maine | North Dakota |  |
| Minnesota | Oregon |  |
| Mississippi | Rhode Island |  |
| Missouri | Utah |  |
| Nebraska | Vermont |  |
| New Hampshire | Washington |  |
| New Jersey |  |  |
| New Mexico |  |  |
| North Carolina |  |  |
| Ohio |  |  |
| Oklahoma |  |  |
| Pennsylvania |  |  |
| South Carolina |  |  |
| Tennessee |  |  |
| Texas |  |  |
| West Virginia |  |  |
| Wisconsin |  |  |
| Wyoming |  |  |

Source: Harlow, et al. Supplement 1. 2024 [9].

| **Appendix 3. Categorization of States by Status of State Legislation on Cannabis (Delta-9 THC)** | | |
| --- | --- | --- |
| **Cannabis Use Legalization Status ^a^** | | |
| **Cannabis Use Illegal** | **Cannabis Use Legal** | **Transition States** |
| Georgia | Alaska | Alabama |
| Idaho | Arizona | Mississippi |
| Indiana | Arkansas | South Dakota |
| Iowa | California |  |
| Kansas | Colorado |  |
| Kentucky | Connecticut |  |
| Nebraska | Delaware |  |
| North Carolina | District of Columbia |  |
| South Carolina | Florida |  |
| Tennessee | Hawaii |  |
| Texas | Illinois |  |
| Wisconsin | Louisiana |  |
| Wyoming | Maine |  |
|  | Maryland |  |
|  | Massachusetts |  |
|  | Michigan |  |
|  | Minnesota |  |
|  | Missouri |  |
|  | Montana |  |
|  | Nevada |  |
|  | New Hampshire |  |
|  | New Jersey |  |
|  | New York |  |
|  | New Mexico |  |
|  | North Dakota |  |
|  | Ohio |  |
|  | Oklahoma |  |
|  | Oregon |  |
|  | Pennsylvania |  |
|  | Rhode Island |  |
|  | Utah |  |
|  | Vermont |  |
|  | Virginia |  |
|  | Washington |  |
|  | West Virginia |  |
| ^a^ National Conference of State Legislatures [17]. See methods for category definitions. | | |

**Appendix 4. Rate of Exposures Involving** **Delta-8 THC Reported to the National Poison Data System by United States Region and by Year, 2021-2022**

**Appendix 5. Rate of Exposures Involving Delta-8 THC Reported to the National Poison Data System by Year and Status of State Regulation of Delta-8 THC, 2021-2022**

**Appendix 6. Rate of Exposures Involving Delta-8 THC Reported to the National Poison Data System by Year and Status of State Cannabis (Delta-9 THC) Legalization, 2021-2022**
