# Supplementary material for: Delta-8 Tetrahydrocannabinol Exposures Reported to US Poison Centers: Variations Among US States and Regions and Associations with Public Policy
Source: J Med Toxicol. 2024 Aug 21;20(4):389–400. doi: 10.1007/s13181-024-01030-z (PMC11436591; doi:10.1007/s13181-024-01030-z)
Supplement: Supplementary file 2 — Supplementary Material 2 [file 13181_2024_1030_MOESM2_ESM.docx]

**D****elta-8 Tetrahydrocannabinol Exposures Reported to US Poison Centers: Variations Among US States and Regions and Associations with Public Policy**

**ABSTRACT**

**Introduction:** This study investigated exposures involving ∆8-tetrahydrocannabinol (∆8-THC) reported to US poison centers (PCs), including variation among states and regions. It evaluated whether the ∆8-THC exposure rate was lower among states with ∆8-THC regulations and states where cannabis (∆9-THC) use was legal.

**Methods:** National Poison Data System data for ∆8-THC exposures in 2021-2022 were analyzed, including comparisons of state and regional population-based exposure rates.

**Results:** There were 4,925 exposures involving ∆8-THC as the primary substance reported to US PCs from January 1, 2021, to December 31, 2022, with 69.8% of these reported in the US South. The rate of exposure per 100,000 US population increased by 79.2% from 0.53 in 2021 to 0.95 in 2022. In 2022, the mean rate of ∆8-THC exposures in states where cannabis use was illegal was 1.64 per 100,000 population (95% CI: 1.08-2.20) compared with 0.52 (95% CI: 0.29-0.76) in states where cannabis use was legal (*P*=0.0010). In 2022, the mean rate of ∆8-THC exposures in states where ∆8-THC was unregulated was 1.36 per 100,000 population (95% CI: 0.95-1.77) compared with 0.17 (95% CI: 0.06-0.27) in states where ∆8-THC was banned (*P*<0.0001).

**Conclusions:** The rate of ∆8-THC exposures reported to US PCs increased by 79% from 2021 to 2022, with the US South accounting for more than two-thirds of exposures. The rate of ∆8-THC exposures reported to PCs was significantly lower among states where ∆8-THC was banned and among states where cannabis use was legal. Consistent regulation of ∆8-THC across all states should be adopted.

**Key words****:** delta-8 tetrahydrocannabinol, tetrahydrocannabinol, public policy, regulation, poisoning

**INTRODUCTION**

The Agriculture Improvement Act (known as the Farm Bill) of 2018 legalized hemp and hemp compounds and derivatives containing <0.3% ∆9-tetrahydrocannabinol (∆9-THC), which is the main psychoactive substance in cannabis.[^1^](#_ENREF_1)^,^ [^2^](#_ENREF_2) Following passage of the Farm Bill, hemp production increased by 445%, leading to a hemp surplus and decrease in cannabidiol (CBD) prices.[^3^](#_ENREF_3) These circumstances resulted in a rapid increase in the manufacture of synthetic tetrahydrocannabinol (THC) substances from CBD that have psychoactive effect profiles similar to ∆9-THC.[^3^](#_ENREF_3) One of these substances is ∆8-tetrahydrocannabinol (∆8-THC), which is available in edibles, beverages, vaping products, and other goods widely sold in stores, gas stations, and online, often without minimum age or age verification requirements.[^4-7^](#_ENREF_4) Clinical effects of ∆8-THC include bradycardia, respiratory depression, slurred speech, lethargy, and coma.[^4^](#_ENREF_4)^,^ [^5^](#_ENREF_5)

In a 2023 cross-sectional survey using a national probability sample of United States (US) adults, the prevalence of ∆8-THC use was estimated to be 11.9%.[^8^](#_ENREF_8) Another national survey of twelfth grade students in 2023 found that 11.4% self-reported use of ∆8-THC in the past year, and more than one-third of those reported that they used it more than ten times during that period.[^9^](#_ENREF_9) In part, because the manufacturing, labeling, and packaging of ∆8-THC are not regulated by the US Food and Drug Administration, a host of safety issues associated with ∆8-THC products has been documented, including contamination with other cannabinoids, heavy metals, solvents, and pesticides; inaccurate or incomplete product labeling; and lack of child-resistant packaging.[^3^](#_ENREF_3)^,^ [^5-7^](#_ENREF_5)^,^ [^10^](#_ENREF_10)^,^ [^11^](#_ENREF_11) The absence of federal regulatory oversight and the growing popularity and use of ∆8-THC have led 15 states to ban ∆8-THC and an additional 9 states to restrict its use.[^2^](#_ENREF_2)^,^ [^5^](#_ENREF_5)^,^ [^9^](#_ENREF_9)^,^ [^12^](#_ENREF_12)^,^ [^13^](#_ENREF_13)

Despite public health concerns, there has been relatively little research on ∆8-THC,[^5^](#_ENREF_5)^,^ [^14^](#_ENREF_14) including on US state and regional variations and how state regulations may influence the public health impact of ∆8-THC. Two studies have reported on the associations between state regulation of ∆8-THC or ∆9-THC and self-reported ∆8-THC use; however, their study designs had limitations related to potential bias and poor generalizability.[^8^](#_ENREF_8)^,^ [^9^](#_ENREF_9) One of these studies included only twelfth grade students in a sample that did not include all US states,[^9^](#_ENREF_9) and the other study was a survey of adults 18 years or older (median age: 48; interquartile range: 33-63 years) with a completion rate of 17.5%.[^8^](#_ENREF_8) The objective of our study was to investigate exposures involving ∆8-THC reported to US poison centers (PCs), with an emphasis on state and regional variations. We hypothesized that the rate of reported ∆8-THC exposures was lower among states with ∆8-THC regulations and among states where cannabis (∆9-THC) use was legal.

**METHODS**

**Data Sources**

Data from the National Poison Data System (NPDS) were analyzed in this retrospective observational study. The NPDS is maintained by America’s Poison Centers and comprises data from calls to regional PCs that are uploaded in near real-time. Product codes for ∆8-THC were introduced into the NPDS in late 2020. Population estimates for 2021 and 2022 were obtained from the US Census Bureau and were used to calculate ∆8-THC exposure rates (including state-specific and US region-specific exposure rates) per 100,000 US population. This study was determined to be exempt from approval by the institutional review board at the authors’ institution.

**Inclusion and Exclusion Criteria**

This study included exposures involving ∆8-THC as the primary substance reported to the NPDS from the 50 US states and District of Columbia from January 1, 2021, through December 31, 2022. The primary substance is the substance that was most likely to be responsible for the observed clinical effects, based on the judgment of Specialists in Poison Information at a PC. Exposures with a medical outcome of “confirmed non-exposure” or a reason for exposure identified as “adverse reaction - food” were excluded from the study. One fatality involving ∆8-THC was excluded from analyses by consensus reached by our study team because it was unlikely to be related to the death based on information included in the de-identified case narrative provided by America’s Poison Centers from the reporting PC. It involved the single-substance ingestion of a ∆8-THC gummy two days prior to contacting the PC and hospital admission, and NPDS records indicated that all clinical effects were "unknown if related" to ∆8-THC.

**Variables**

Study variables included year, age group, sex, route of exposure (ingestion, inhalation, or other), reason for exposure, exposure type (single-substance or multiple-substance exposure), US region (Northeast, Midwest, South, and West) [^15^](#_ENREF_15) (Appendix 1), highest level of health care received, and medical outcome. Age groups were categorized as 1) <6 years (young children), 2) 6-19 years (children/teenagers), 3) 20-59 years (adults), and 4) >59 years (older adults). We used the NPDS^[16](#_ENREF_16)^ categories for reason for exposure: 1) unintentional-general, 2) unintentional-other (includes environmental/occupational/misuse), 3) unintentional-unknown, 4) intentional-suspected suicide, 5) intentional-misuse, 6) intentional-abuse, 7) intentional-unknown, 8) other, and 9) unknown.

We also used the NPDS[^16^](#_ENREF_16) categories for highest level of health care received:1) no healthcare facility (HCF) treatment received, 2) treated/evaluated and released, 3) admitted to a critical care unit (CCU), 4) admitted to a non-CCU, 5) admitted to a psychiatric facility, 6) patient refused referral/did not arrive at a HCF, or 7) patient lost to follow-up/left against medical advice/unknown. Exposures with management site coded as “unknown” were included in the “lost to follow-up/left against medical advice/unknown” category, and this category was considered as unknown during analyses.

We analyzed medical outcomes as defined by the NPDS[^16^](#_ENREF_16): 1) no effect, 2) minor effect (minimal symptoms that generally resolve rapidly), 3) moderate effect (more pronounced, prolonged, or systemic symptoms than minor effect), 4) major effect (symptoms are life-threatening or result in significant disability or disfigurement), 5) death, 6) not followed (minimal clinical effects possible), 7) unrelated effect, or 8) unable to follow (judged as potentially toxic exposure). The category, “unable to follow (judged as potential toxic exposure),” was considered unknown during analyses.

Consistent with the categorization by Harlow, et al.,[^9^](#_ENREF_9) the 50 US states and District of Columbia were grouped according to the status of their ∆8-THC regulations prior to January 1, 2023, which yielded three groups: 1) ∆8-THC unregulated by the state, 2) ∆8-THC banned by the state, and 3) ∆8-THC restricted (but not banned) by the state, including restriction of legal use to individuals 21 years and older and banning products containing >0.3% of any form of THC (Appendix 2). In addition, based on the categories used by the National Conference of State Legislatures,[^17^](#_ENREF_17) the status of cannabis (∆9-THC) legalization for the 50 US states and District of Columbia was stratified into six groups: 1) cannabis use is illegal, 2) low ∆9-THC, high CBD product use is legal, 3) only medical cannabis use is legal, 4) transitioning to legal medical cannabis use, 5) transitioning from legal medical cannabis use to legal recreational cannabis use, and 6) both medical and recreational cannabis use is legal. The definition of “low ∆9-THC, high CBD product” varies among state laws. In this study, “transitioning” to a new cannabis legalization status means that a new law went into effect during the study period. For analyses, these six groups were combined into the categories: 1) “cannabis use illegal,” which included states where cannabis use was illegal and states where only low ∆9-THC, high CBD product use was legal and 2) “cannabis use legal,” which included states where medical cannabis use was legal, states transitioning from legal medical cannabis to legal recreational cannabis use, and states where both medical and recreational cannabis use was legal. States that were transitioning to legal medical cannabis use were grouped in a third category, called "transition states," that represented states moving from illegal to legal medical cannabis use during the study period. This transition category was used in sensitivity analyses (Appendix 3).

**Statistical Analysis**

Statistical analyses were performed using SAS 9.4 (SAS Institute, Inc. Cary, North Carolina) and IBM SPSS Statistics 28.0 (IBM Corporation, Armonk, New York) software. Initially, descriptive statistics were used to characterize the data prior to performing inferential statistics. ∆8-THC exposure rates were compared between groups of states based on their ∆8-THC regulation status and their ∆9-THC legalization status. First, the mean rate of exposure to Δ8-THC per 100,000 US population was compared between states where Δ8-THC was unregulated versus states where it was banned using the Mann Whitney U-test for each year in our study. Sensitivity analyses were performed by repeating the comparisons while adding states where Δ8-THC was restricted to either the unregulated states or states that banned Δ8-THC. Second, the mean rate of exposure to Δ8-THC per 100,000 US population was compared between states where cannabis (Δ9-THC) use was illegal versus states where it was legal during the study period using the Mann-Whitney U-test for each year in our study. Sensitivity analyses were performed by repeating the comparisons while adding transition states to either the cannabis illegal or cannabis legal categories. The non-parametric Mann-Whitney U-test was used because the mean rates of exposure from these independent samples of states were not normally distributed based on the Shapiro-Wilk test for normality. The level of significance for all comparisons was a=0.05.

**RESULTS**

There were 4,925 exposures involving ∆8-THC as the primary substance reported to US PCs during 2021 and 2022. The number of exposures increased by 82.1% from 1,746 exposures in 2021 to 3,179 in 2022, and the rate of ∆8-THC exposures per 100,000 US population increased by 79.2% from 0.53 in 2021 to 0.95 in 2022. The 20-59-year-old age group accounted for 40.5% of exposures, followed by <6-year-olds (30.4%) and 6-19-year-olds (24.4%). Most cases were single-substance exposures (94.3%) or ingestions (94.3%) (Table 1). The reason for exposure was most commonly unintentional-general (39.8%), followed by abuse (33.3%); however, this varied by age with the unintentional-general category accounting for 99.2% of exposures among <6-year-olds and intentional reasons accounting for most exposures among older age groups. Most ∆8-THC exposures (52.4%) were treated/evaluated and released, although 15.5% were admitted to either a non-CCU or CCU. Children <6 years old accounted for 50.0% of non-CCU admissions and 57.8% of CCU admissions, as well as 27.7% of moderate and 32.5% of major medical outcome effects. Individuals most commonly experienced the medical outcomes of minor effect (38.6%), followed by moderate effect (35.6%) and major effect (2.9%) in association with ∆8-THC exposures. The one reported fatality was a 2-year-old boy with a single-substance, unintentional-general exposure. A case narrative was requested, but not available, from the reporting PC, and the relative contribution to fatality determination awaits completion of review by the America’s Poison Centers.

**Variations among US States and Regions**

Figure 1 illustrates the variation in ∆8-THC exposure rates among states and the increases in rates from 2021 to 2022. There were exposures reported from every state and the District of Columbia during the study period. In 2021, South Dakota was the state with the highest exposure rate per 100,000 population (2.12), followed by Minnesota (2.00), Alabama (1.90), and West Virginia (1.90). In 2022, Alabama was the state with the highest exposure rate (3.35), followed by South Dakota (3.19), Tennessee (2.82), and West Virginia (2.82). More than two-thirds (69.8%) of reports to US PCs involving ∆8-THC were in the South, followed by the Midwest (22.2%), Northeast (5.8%), and West (2.2%) (Table 2). Adults 20-59 years old accounted for the most reported exposures in the South (41.1%) and Midwest (42.4%) regions, while <6-year-olds accounted for the most exposures in the West (41.4%). Ingestions accounted for most exposures in all regions (95.6% South, 92.5% Midwest, 95.1% Northeast, and 91.6% West). More than half of the exposures (70.5%) associated with ingestion were reported in the South, followed by the Midwest (21.6%) (Table 2). Abuse was the most common reason for exposure in the Midwest (40.4%), whereas unintentional-general was the most common in the other regions (Northeast 43.2%, South 41.3%, and West 50.5%).

The proportion of individuals admitted to either a non-CCU or CCU in association with a reported ∆8-THC exposure was lower in the West (6.6%) than in the Midwest (15.7%), Northeast (13.4%), or South (15.9%) regions. Among medical outcomes, individuals experiencing moderate effects were proportionately less common in the West (22.0%) than in the Midwest (32.4%), Northeast (34.5%), or South (37.3%) regions. Individuals experiencing major effects demonstrated a similar pattern, with a slightly lower proportion in the West (1.1%) than in the Midwest (2.5%), Northeast (2.6%), or South (3.1%) regions (Table 2).

The rate of ∆8-THC exposures per 100,000 US population increased in all four US regions from 2021 to 2022, with the highest rate observed in the South, followed by the Midwest (Appendix 4). However, the greatest percentage increase in the rate occurred in the West (100.0%; 0.01 in 2021 to 0.02 in 2022) and the Northeast (100.0%; 0.03 in 2021 to 0.06 in 2022), followed by a 90.9% increase in the Midwest (0.11 in 2021 to 0.21 in 2022) and a 71.1% increase in the South (0.38 in 2021 to 0.65 in 2022).

**State Comparisons Based on ∆8-THC Regulation Status**

In 2022, the mean rate of exposures involving ∆8-THC in states where ∆8-THC was unregulated was 1.36 per 100,000 population (95% CI: 0.95-1.77), with a median of 1.28 (95% CI: 0.53-2.22); the mean rate of exposures in states where ∆8-THC was banned was 0.17 (95% CI: 0.06-0.27), with a median of 0.08 (95% CI: 0.06-0.18). There was a statistically significant difference in the mean rate of exposures between states where ∆8-THC was unregulated and states where ∆8-THC was banned (Mann-Whitney, *P*<0.0001). During a sensitivity analysis, there was relatively minor change in these values when the states where ∆8-THC was restricted (but not banned) were added to either the states where ∆8-THC was unregulated or states where ∆8-THC was banned. The analyses involving rates for 2021 did not differ substantially from those for 2022, although the rates in 2021 were lower than in 2022 (Table 3).

The rate of ∆8-THC exposures increased from 2021 to 2022 among all three state groups irrespective of their ∆8-THC regulation status. There was a 77.8% increase in the rate among states where ∆8-THC was unregulated, 200.0% increase among states where ∆8-THC was banned, and 100.0% increase among states where ∆8-THC was restricted (but not banned) (Appendix 5).

**State Comparisons Based on Cannabis (∆9-THC) Legalization Status**

In 2022, the mean rate of exposures involving ∆8-THC in states where cannabis (∆9-THC) use was illegal was 1.64 per 100,000 population (95% CI: 1.08-2.20), with a median of 1.88 (95% CI: 0.34-2.46); the mean rate of ∆8-THC exposures in states where cannabis use was legal was 0.52 (95% CI: 0.29-0.76), with a median of 0.21 (95% CI: 0.14-0.53). There was a statistically significant difference in the mean rate of exposures between states where cannabis use was illegal and states where cannabis use was legal (Mann-Whitney, *P*=0.0010). During a sensitivity analysis, there was relatively minor change in these values when transition states were added to either group of states where cannabis use was illegal or legal. The analyses involving rates for 2021 did not differ substantially from those for 2022, although the rates in 2021 were lower than in 2022 (Table 4).

The rate of ∆8-THC exposures increased from 2021 to 2022 for each of the three groups of states based on cannabis legalization status, with the greatest increase (100.0%) seen among transition states, followed by 78.6% in states where cannabis use was illegal and 76.2% in states where cannabis use was legal (Appendix 6).

**DISCUSSION**
The US Drug Enforcement Administration issued an interim final rule in August 2020 to clarify the Farm Bill, indicating that ∆8-THC and other synthetically derived tetrahydrocannabinols were Schedule I controlled substances; however, there continues to be widespread sale and use of ∆8-THC.[^4^](#_ENREF_4)^,^ [^18^](#_ENREF_18)^,^ [^19^](#_ENREF_19) Our study demonstrated a 79% increase in the rate of reported ∆8-THC exposures to US PCs from 2021 to 2022. Although most ∆8-THC exposures (52%) were treated/evaluated and released, a notable 16% were admitted to either a non-CCU or CCU. ∆8-THC exposures were commonly associated with a minor effect (39%) or moderate effect (36%), with a minority experiencing a major effect (3%).

Although ∆8-THC products are intended for use by adults, children represented more than half of exposures (30% were <6 years old and 24% were 6-19 years old), and children <6 years old accounted for half of non-CCU admissions and 58% of CCU admissions, as well as 28% of moderate and approximately one-third of major medical outcomes. The high proportion of hospital admissions and serious medical outcomes among young children may be attributable, in part, to the relatively greater ∆8-THC dose per body weight among pediatric exposures compared with adult exposures.[^20^](#_ENREF_20)^,^ [^21^](#_ENREF_21) This is likely exacerbated by young children mistaking edible ∆8-THC products for food or candy, the presence of multiple doses in one product package (some totaling hundreds of milligrams), and the delay in onset of clinical effects that allows continued consumption of the product before the child or caregivers notice that something is wrong.[^21^](#_ENREF_21)^,^ [^22^](#_ENREF_22) In addition, minors can readily access ∆8-THC, which is often marketed in ways that appeal to teenagers, can often be obtained without age verification, and typically costs less than ∆9-THC.[^6^](#_ENREF_6)^,^ [^7^](#_ENREF_7)^,^ [^23^](#_ENREF_23)

The number and characteristics of ∆8-THC exposures reported to PCs varied widely by US region. The South accounted for 70% of exposures, followed by the Midwest (22%), while the Northeast and West represented only 6% and 2%, respectively. These findings are consistent with those of a survey of selected twelfth grade students, which found that reported ∆8-THC use was higher in the South and Midwest.[^9^](#_ENREF_9) The age distribution of exposed individuals and the reason for exposure also varied by region in our study, with children <6 years old and the exposure reason “unintentional – general” (which represents exploratory behavior in this age group) more common in the West, while 20-59-year-olds and “abuse” more common in the Midwest and South. The proportion of exposures that were admitted to a non-CCU or CCU was lower in the West than in other regions. Likewise, the proportion of exposures associated with moderate or major effects was lower in the West than in other regions. The reasons for these variations cannot be answered by this study but represent areas of future research. Prevention strategies are different for exposures among young children associated with exploratory behavior than intentional exposures among teenagers and adults, and findings from this study suggest that target populations and the types of population-based prevention interventions may need to vary by region.

Cannabis (∆9-THC) is the most commonly used illicit drug in the world;[^24^](#_ENREF_24) however, legal access to cannabis for recreational use has increased rapidly in the US with changes in state laws during recent years. As hypothesized, our findings showed a statistically significant lower rate of ∆8-THC exposures reported to PCs among states where medical or recreational cannabis use was legal than states where cannabis use was illegal. This is likely attributable, in part, to less market competition from ∆9-THC products in states where their use was illegal, and that ∆8-THC was likely being used as a substitute for ∆9-THC. This is consistent with studies that found a higher proportion of internet queries about ∆8-THC in states where recreational cannabis was illegal than in states where it was legal.[^25^](#_ENREF_25)^,^ [^26^](#_ENREF_26) This is also consistent with research that showed greater self-reported ∆8-THC use among surveyed twelfth grade students in states without cannabis legislation[^9^](#_ENREF_9) and lower reported use of ∆8-THC by adults who lived in states with medical or recreational cannabis laws.[^8^](#_ENREF_8)

In addition, as hypothesized, there was a statistically significant lower rate of ∆8-THC exposures reported to PCs among states where ∆8-THC was banned than states where it was unregulated. This reflects the potential for regulation to reduce potentially harmful exposures and is consistent with the findings from a survey of selected twelfth grade students, who reported higher ∆8-THC use prevalence in states without ∆8-THC regulations.[^9^](#_ENREF_9) Although, to-date, public policy efforts have focused more on ∆9-THC, our study’s findings support the need for adoption of consistent regulation of ∆8-THC across all states.

**Study Limitations**This study has several limitations. This study underestimates the number of ∆8-THC exposures because not all these exposures are reported to US PCs, rather, they may be cared for in emergency departments or other healthcare settings without PC involvement, or not require health care at all. Reporting to a PC may be biased related to such factors as severity or age. As in any large database, miscoding may occur. The NPDS contains self-reported data that may not be completely verified by the PCs or America’s Poison Centers, and many of the individuals in this study did not receive a medical evaluation. Reported exposures do not necessarily represent a poisoning or overdose. Our analyses did not take into account that some states have both ∆8-THC regulations as well as legalization of cannabis when analyzing the associations of these policies with the rate of ∆8-THC exposures reported to PCs. Because of inadequate quality control and labeling, other cannabinoids and adulterants may be present in the involved ∆8-THC products. Even if toxicological testing were done, results were not included in the database. The clarity or prominence of the labeling of ∆8-THC products, HCF and PC staff familiarity with these products, and willingness of non-HCF individuals to identify ∆8-THC products may vary by state based on the legal status of ∆8-THC; this may account for some of the observed variations by state. Because product codes for ∆8-THC were introduced into the NPDS in late 2020, increasing familiarity with the new codes may have contributed to the observed increase in reported exposures. In addition, two years of data cannot truly define a trend, so this awaits future study. Despite its limitations, the NPDS database is useful for investigating the patterns of ∆8-THC exposures among US states and regions and associations with state regulations.

**CONCLUSIONS**

The rate of ∆8-THC exposures reported to US PCs increased 79% from 2021 to 2022. with the South accounting for more than two-thirds of exposures. The rate of ∆8-THC exposures reported to PCs was significantly lower among states where ∆8-THC was banned and among states where cannabis (∆9-THC) use was legal. Consistent regulation of ∆8-THC across all states should be adopted.

**Acknowledgments**

None

**REFERENCES**

1. 115th Congress (2017-2018). H.R.2 - Agriculture Improvement Act of 2018 [Internet]. [cited 2024 Apr 04]. Available from: <https://www.congress.gov/bill/115th-congress/house-bill/2/text>.

2. US Food and Drug Administration. FDA regulation of cannabis and cannabis-derived products, including cannabidiol (CBD) [Internet]. [cited 2024 Apr 04]. Available from: <https://www.fda.gov/news-events/public-health-focus/fda-regulation-cannabis-and-cannabis-derived-products-including-cannabidiol-cbd>.

3. Geci M, Scialdone M, Tishler J. The dark side of cannabidiol: The unanticipated social and clinical implications of synthetic Δ8-THC. Cannabis Cannabinoid Res. 2023;8(2):270-82.

4. Babalonis S, Raup-Konsavage WM, Akpunonu PD, Balla A, Vrana KE. Δ8-THC: Legal status, widespread availability, and safety concerns. Cannabis Cannabinoid Res. 2021;6(5):362-5.

5. US Centers for Disease Control and Prevention. Increases in availability of cannabis products containing delta-8 THC and reported cases of adverse events. [Internet]. 2021 [cited 2024 Apr 04]. Available from: <https://emergency.cdc.gov/han/2021/pdf/CDC_HAN__451.pdf>.

6. Leas EC. The hemp loophole: A need to clarify the legality of delta-8-THC and other hemp-derived tetrahydrocannabinol compounds. Am J Public Health. 2021;111(11):1927-31.

7. LoParco CR, Rossheim ME, Walters ST, Zhou Z, Olsson S, Sussman SY. Delta-8 tetrahydrocannabinol: A scoping review and commentary. Addiction. 2023;118(6):1011-28.

8. Wilson-Poe AR, Smith T, Elliott MR, Kruger DJ, Boehnke KF. Past-year use prevalence of cannabidiol, cannabigerol, cannabinol, and Δ8-tetrahydrocannabinol among US adults. JAMA Netw Open. 2023;6(12):e2347373.

9. Harlow AF, Miech RA, Leventhal AM. Adolescent delta-8-THC and marijuana use in the US. JAMA. 2024;33(10):861-5.

10. Holt AK, Poklis JL, Peace MR. ∆8-THC, THC-O acetates and CBD-di-O acetate: Emerging synthetic cannabinoids found in commercially sold plant material and gummy edibles. J Anal Toxicol. 2022;46(8):940-8.

11. Ray CL, Bylo MP, Pescaglia J, Gawenis JA, Greenlief CM. Delta-8 tetrahydrocannabinol product impurities. Molecules. 2022;27(20):6924.

12. Kruger DJ, Amila K, Kaplan SM, Redfield J, Stacy T, Agarwal V, Faqqouseh M, Bone CC. A content analysis of social media discussions on THC-O-acetate. Cannabis. 2023;6(2):13-21.

13. Szczypka G, Pepper JK, Kim A. Weed light: An exploratory study of delta-8 THC conversations on Reddit. RTI Press. 2022;RTI Press Research Brief No. RB-0030-2206.

14. Whitehill JM, Dunn KE, Johnson RM. The public health challenge of delta-8 THC and derived psychoactive cannabis products (Editorial). JAMA. 2024;331(10):834-6.

15. US Census Bureau. Census regions and divisions of the United States. [Internet]. 2023 [cited 2024 Apr 04]. Available from: <https://www2.census.gov/geo/pdfs/maps-data/maps/reference/us_regdiv.pdf>.

16. America's Poison Centers. National Poison Data System (NPDS): NPDS coding users’ manual [Internet]. 2023 [cited 2024 Apr 04]. Available from: <https://www.npds.us/Help/NPDS%20Coding%20User%20Manual%20(June%202023).pdf>.

17. National Conference of State Legislature. State medical cannabis laws [Internet]. [cited 2024 Apr 04]. Available from: <https://www.ncsl.org/health/state-medical-cannabis-laws>.

18. US Department of Justice, United States Drug Enforcement Administration. Implementation of the Agriculture Improvement Act of 2018. Federal Register [Internet]. 2020 [cited 2024 Apr 04]. Available from: <https://www.govinfo.gov/content/pkg/FR-2020-08-21/pdf/2020-17356.pdf>.

19. US Department of Justice, United States Drug Enforcement Administration. Controlled Substance Schedules [Internet]. [cited 2024 Apr 15]. Available from: <https://www.deadiversion.usdoj.gov/schedules/orangebook/c_cs_alpha.pdf>.

20. Pepin LC, Simon MW, Banerji S, Leonard J, Hoyte CO, Wang GS. Toxic tetrahydrocannabinol (THC) dose in pediatric cannabis edible ingestions. Pediatrics. 2023;152(3):e2023061374.

21. Tweet MS, Nemanich A, Wahl M. Pediatric edible cannabis exposures and acute toxicity: 2017-2021. Pediatrics. 2023;151(2):e2022057761.

22. Ompad DC, Snyder KM, Sandh S, Hagen D, Collier KJ, Goldmann E, Goodman MS, Tan ASL. Copycat and lookalike edible cannabis product packaging in the United States. Drug Alcohol Depend. 2022;235:109409.

23. Firth S. Lax regulation, colorful packaging, draw minors to delta-8 cannabinoids online [Internet]. MedPage Today; [cited 2024 Apr 04]. Available from: <https://www.medpagetoday.com/meetingcoverage/aap/106950>.

24. World Health Organization. Alcohol, drugs and addictive behaviors unit. Cannabis [Internet]. [cited 2024 Apr 04]. Available from: <https://www.who.int/teams/mental-health-and-substance-use/alcohol-drugs-and-addictive-behaviours/drugs-psychoactive/cannabis>.

25. Leas EC, Nobles AL, Shi Y, Hendricksona E. Public interest in ∆8-tetrahydrocannabinol (delta-8-THC) increased in US states that restricted ∆9-tetrahydrocannabinol (delta-9-THC) use. Int J Drug Policy. 2022;101:103557.

26. Livingston MD, Walker A, Cannell MB, Rossheim ME. Popularity of delta-8 THC on the internet across US states, 2021. Am J Public Health. 2022;112(2):296-9.

**TABLE TITLES**

**Table 1.** Characteristics of Exposures Involving Delta-8 THC Reported to the National Poison Data System by Age Group, 2021-2022

**Table 2.** Characteristics of Exposures Involving Delta-8 THC Reported to the National Poison Data System by United States Region 2021-2022

**Table 3.** Comparison of Exposure Rates Involving Delta-8 THC Reported to the National Poison Data System by the Status of State Regulation of Delta-8 THC and by Year, 2021-2022

**Table 4.** Comparison of Exposure Rates Involving Delta-8 THC Reported to the National Poison Data System by the Status of State Legislation on Cannabis (Delta-9 THC) Use and by Year, 2021-2022

**FIGURE TITLES**

**Figure 1.** Rates of Exposures Involving Delta-8 THC Reported to the National Poison Data System by State for 2021 and 2022

**APPENDIX TITLES**

**Appendix 1.** Categorization of States by United States Region

**Appendix 2.** Categorization of States by Status of State Regulation of Delta-8 THC

**Appendix 3.** Categorization of States by Status of State Legislation on Cannabis (Delta-9 THC)

**Appendix 4.** Rate of Exposures Involving Delta-8 THC Reported to the National Poison Data System by United States Region and by Year, 2021-2022

**Appendix 5.** Rate of Exposures Involving Delta-8 THC Reported to the National Poison Data System by Year and Status of State Regulation of Delta-8 THC, 2021-2022

**Appendix 6.** Rate of Exposures Involving Delta-8 THC Reported to the National Poison Data System by Year and Status of State Cannabis (Delta-9 THC) Legalization, 2021-2022
